# Vancomycin-Resistant Enterococcus Endocarditis Complicated by Splenic Infarction and Embolic Stroke

**DOI:** 10.7759/cureus.40633

**Published:** 2023-06-19

**Authors:** Shivam Khatri, Adisalem Teferi, Simon Kashfi, Salomon Chamay, Shorabh Sharma

**Affiliations:** 1 Medicine, The City University of New York School of Medicine, New York, USA; 2 Internal Medicine, St. Barnabas Hospital Health System, Bronx, USA; 3 Internal Medicine, Donald and Barbara Zucker School of Medicine at Hofstra/Northwell, Hempstead, USA

**Keywords:** sepsis, splenic infarction, embolic stroke, vancomycin resistant enterococcus (vre), infective endocarditis

## Abstract

Infective endocarditis (IE) is a serious condition associated with high morbidity and mortality rates. The risk factors for IE include underlying heart disease, intravenous drug use, cardiac surgery, and interventional procedures. Enterococci are a common cause of IE, and vancomycin-resistant enterococci (VRE) infections are becoming increasingly prevalent. In this report, we present the case of an 88-year-old female patient with multiple cardiac comorbidities who developed VRE endocarditis with splenic infarction and embolic stroke. The patient was successfully treated with a combination of antibiotics and anticoagulation therapy. This report highlights the importance of recognizing the potential complications of VRE endocarditis and the need for appropriate management to prevent adverse outcomes. To the best of our knowledge, only one other case of VRE endocarditis with multiple systemic complications has been documented so far.

## Introduction

Infective endocarditis (IE), defined by a focus of infection within the endocardial surface of the heart, is associated with a high risk of morbidity and mortality, with its prognosis highly dependent on complications [1,2]. In the United States, IE occurs in about one per 33,000 people, predominantly affecting males (50-65% of patients), with an average age at presentation between 52-63 years. The major risk factors for IE include underlying heart disease, intravenous drug use, cardiac surgery and interventional procedures, and prosthetic valves, among others [3]. The most common organisms that cause IE are staphylococci and streptococci. Enterococci are the third leading cause of IE and are seen in about 5-20% of cases, and are often resistant to vancomycin. Vancomycin-resistant enterococci (VRE) faecalis endocarditis is associated with central venous lines, liver transplantation, and mitral valve infections, whereas VRE faecium endocarditis is most commonly associated with infection of the tricuspid valve [4].

What makes IE so dangerous is the fact that it may embolize to major arterial beds including but not limited to the spleen, kidney, liver, and brain [5]. One study involving 493 patients with IE demonstrated that septic emboli were present in 57% of patients and they complicated about 20-50% of cases of IE of left-sided heart valves. These numbers may be higher in those with cardiac valve replacement [6]. In fact, embolism to the spleen can result in splenic infarction in 40% of patients, with splenic abscess occurring in 5% of these patients [7]. Although there are multiple case reports that involve VRE endocarditis leading to splenic infarction, the presence of VRE with multiple systemic complications has seldom been reported and its medical management is not as well documented. We present a case of an 88-year-old female patient with multiple cardiac comorbidities, including acquired aortic stenosis status post-transcatheter aortic valve replacement (TAVR), who developed VRE endocarditis with splenic infarct and embolic stroke.

## Case presentation

An 88-year-old Caucasian female with a history of hypertension, dyslipidemia, hypothyroidism, aortic stenosis status post-bioprosthetic TAVR two years and four months ago, coronary artery disease, congestive heart failure with preserved ejection fraction (HFpEF) and permanent atrial fibrillation, was brought in by emergency medical services (EMS) after her neighbors noticed that they had not heard from her for over a day. EMS had found her lying on the floor. Her home medications included metoprolol, simvastatin, and apixaban. The patient had no history of smoking or other illicit drug use.

In the emergency department (ED), the patient had an initial temperature of 98.5 °F, a heart rate of 86 beats per minute, and a blood pressure of 140/67 mmHg. On physical examination, the patient was alert, oriented to person and place, and obeying some commands. She also had dry mucous membranes, an irregular heartbeat, some bruising over the left buttocks, and suprapubic tenderness. On laboratory workup, she was found to have elevated lactate levels and non-specific ECG changes with an elevated troponin. Relevant labs are shown in Tables 1, 2.

**Table 1:** Complete blood count in the ED ED: emergency department

Test	Reference range (units)	Result
White blood cells	4.0-10 (10^3^/uL)	8.2
Hemoglobin	11.2-15.7 (g/dl)	9.5
Platelet	150-450 (10^3^/uL)	92

**Table 2:** Comprehensive metabolic panel in the ED ED: emergency department

Variables	Reference range (units)	Result
Sodium	135-145 meq/L	137
Potassium	3.5-5.3 meq/L	4.0
Chloride	96-108 meq/L	102
Glucose	70-99 mg/dl	173
Calcium	9.2-11 mg/dl	8.9
Urea nitrogen	8-23 mg/dl	41
Creatinine	0.6-1.2 mg/dl	1.2
Total protein	6.0-8.0 g/dl	6.3
Total bilirubin	0.1-1.2 mg/dl	3.7
Aspartate aminotransferase	9-33 U/L	115
Alanine transaminase	4-36 U/L	47
Alkaline phosphatase	38-126 U/L	81
Creatinine phosphokinase	26-140 IU/L	3590
Lactate	0.0-2.0 mmol/L	2.3
Troponin I	0.0-0.49 ng/ml	1.18

The patient was admitted due to non-ST-elevation myocardial infarction (NSTEMI) and rhabdomyolysis. Cefepime and vancomycin were initiated for empiric coverage on admission. The elevated troponin was attributed to demand ischemia and she did not require further cardiac intervention. Urine cultures collected in the ED were negative, while initial blood cultures on the blood culture identification (BCID) molecular test were positive for Enterococcus faecalis without Van A/B genes depicting vancomycin sensitivity. However, the Enterococcus species could not be isolated on the actual final blood culture result, which was concerning for a possible false-positive BCID result. A repeat blood culture after two days also did not grow the species. A transthoracic echocardiogram (TTE) was performed, which revealed severe mitral annular calcifications but no evidence of vegetation. A transesophageal echocardiogram (TEE) was not performed because the patient was not a surgical candidate due to her underlying medical conditions and the wishes of the patient and her family that there be no surgical interventions. Based on the above considerations, antibiotic therapy was continued with IV ampicillin to empirically cover the Enterococcus species identified on BCID despite the negative growth on culture.

**Figure 1:**
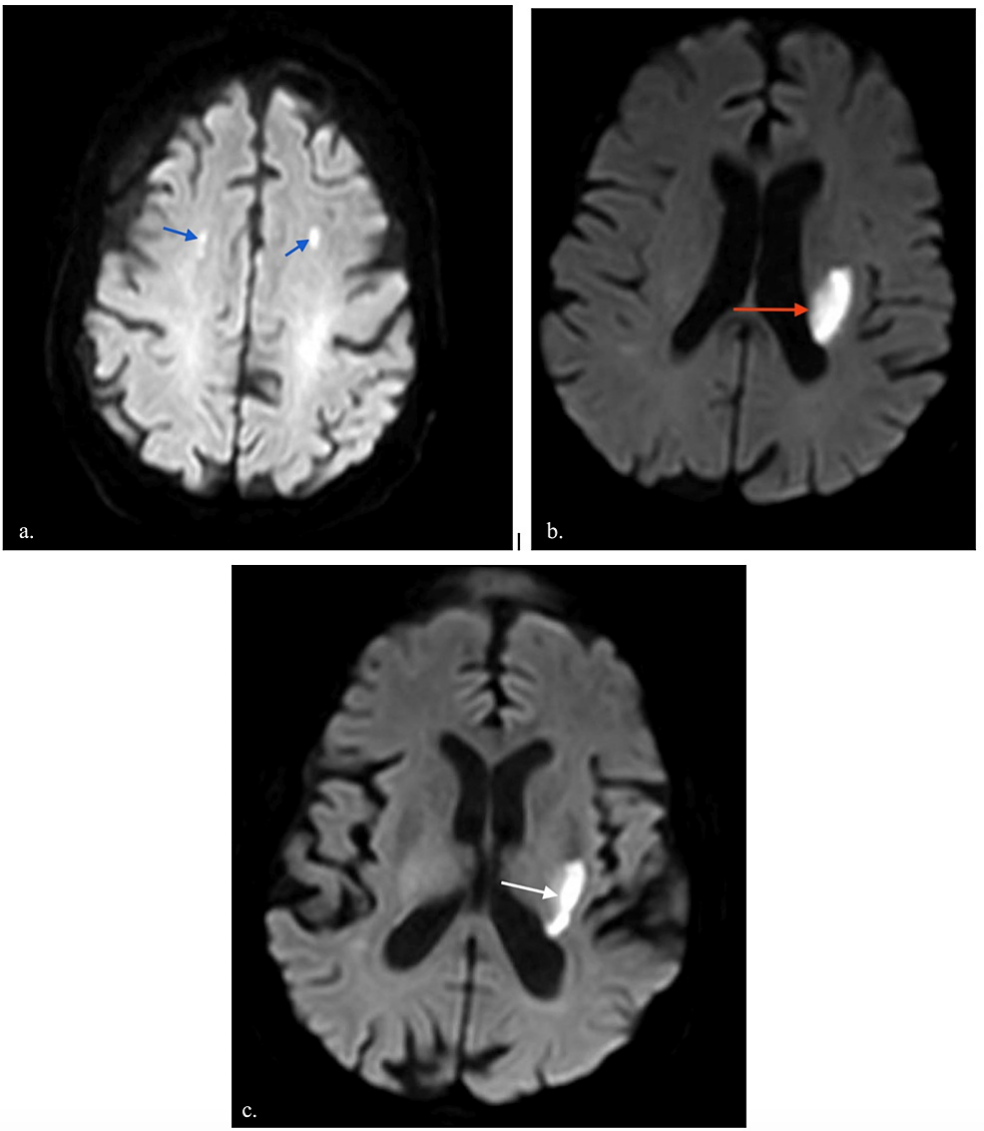
MRI of the brain (a) Diffusion-weighted sequence imaging demonstrated punctate foci of restricted diffusion deep in the white matter of the cerebral hemispheres (blue arrows). (b) There were also restricted diffusion patterns seen in the posterior half of the left internal capsule (red arrow) and the (c) external capsule (white arrow). These widespread patterns indicated embolic events spanning multiple cerebral vascular territories suggesting the showering of emboli consistent with ischemic stroke from a central embolic source, in this case from cardiac valve vegetations MRI: magnetic resonance imaging

Although the patient's condition improved gradually over the following days, she remained hospitalized for several more days to undergo a repeat echocardiogram and to optimize her renal function. Twelve days after admission, the patient developed slurred speech with weakness in the right upper and lower extremities. There was a decreased sensation to pinprick on her right face with right-sided upper motor neuron facial palsy. An urgent CT scan of the brain showed a lacunar infarction of the left corona radiata. The patient was considered to be at a high bleeding risk by the primary team because of her advanced age and current anticoagulant therapy, and hence thrombolytic therapy was deferred. MRI of the brain revealed acute bilateral white matter infarcts spanning multiple vascular territories consistent with a central embolic source (Figure 1).

**Figure 2:**
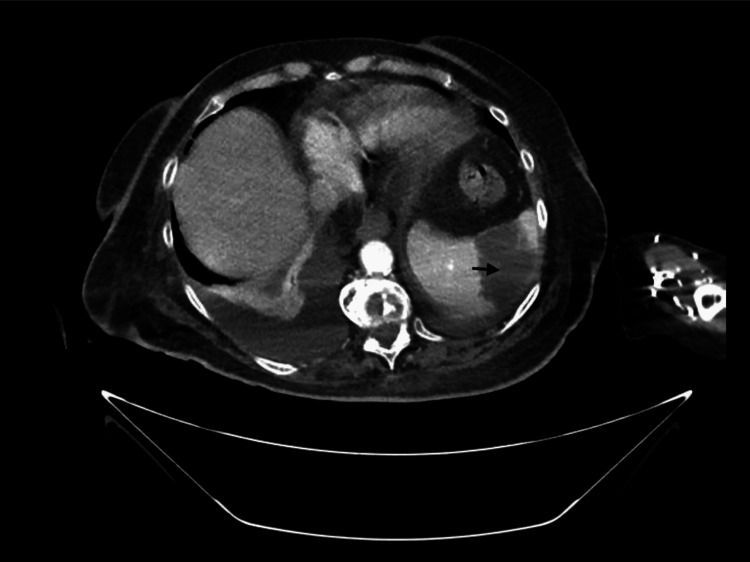
Contrast-enhanced abdominal CT scan demonstrates a triangular area of hypodensity (arrow) in the spleen consistent with splenic infarction CT: computed tomography

The TTE study showed severe mitral annular calcifications with mobile echo densities on the valve suggestive of vegetations. This was associated with moderate mitral regurgitation. The bioprosthetic aortic valve was found to have normal function, with no report of regurgitation or stenosis. In addition, the patient now complained of left upper quadrant abdominal pain for which a workup with an abdominal CT scan was performed. The scan showed a 5.1 cm splenic hypodensity consistent with splenic infarction. This was most likely caused by the embolization of the mitral valve vegetation (Figure 2).

Cardiothoracic surgery was consulted and they opined the patient was not a surgical candidate due to her age and functional status. Hence, further imaging with a TEE was deferred and it was recommended to continue medical treatment with antibiotics. Repeat blood cultures collected three days after the patient developed a stroke were positive for Enterococcus faecium identified both on the BCID molecular panel and actual culture. This strain was found to be resistant to vancomycin, daptomycin, and ampicillin, while sensitive to both linezolid and tigecycline. The patient was treated with antibiotics for vancomycin-resistant Enterococcus with intravenous (IV) linezolid. Later, her antibiotics regimen was changed to IV eravacycline and ampicillin for a total of six weeks. This latter antibiotic choice was based on the susceptibility of the bacterial strain to tigecycline, and ampicillin was added for synergistic effect based on Infectious diseases (ID) recommendations. After 72 hours from the VRE culture result, repeat blood cultures yielded negative results. The patient was discharged to a skilled nursing facility while on IV medications. Her neurological deficits did not improve on antibiotic therapy. Three weeks following her initial discharge, the patient was readmitted to the medical ICU due to septic shock caused by a urinary tract infection attributed to Candida. Unfortunately, the patient passed away after a brief hospital stay. A repeat TTE conducted during this readmission did not reveal any vegetation.

## Discussion

IE with cerebral stroke and splenic infarction remains a complex clinical scenario. A septic embolism occurs when a large bacterial inoculum forms on the vulnerable vascular territory and the vegetation dislodges into smaller emboli that travel through the bloodstream and occlude individual blood vessels [6]. The spleen is particularly vulnerable to infarction due to its lack of collateral circulation. Splenic infarction occurs in approximately 40% of left-sided IE cases, with 90% of splenic infarcts and abscesses having no symptoms [1]. Neurological complications of embolic IE may also occur and include stroke, transient ischemic attack, meningitis, and intracranial hemorrhage. In fact, the central nervous system is the most common site for embolic events with an incidence rate of up to 65% [1]. It would have been commonplace if this patient was diagnosed with a single embolic complication of IE; however, the finding of a splenic infarct and stroke in the presence of VRE endocarditis makes this case relatively unusual, and management in such cases is not well-established. As stated earlier, a TTE was performed and was suggestive of vegetation; however, a TEE was not pursued. Upon further consultation with microbiology, the initial positive BCID result for the vancomycin-sensitive strain of Enterococcus faecalis was very likely to have been false-positive given the fact that no Enterococcus species were isolated on the actual culture despite the positive molecular identification. However, subsequent blood cultures following the patient's embolic stroke definitively tested positive for Enterococcus faecium both on the BCID panel as well as on the actual final culture result.

Lindsey et al. reported a case of a 75-year-old male who presented with VRE faecalis aortic valve endocarditis that led to embolic stroke and splenic abscess. Their patient was treated with emergent valve replacement followed by delayed robotic splenectomy [7]. In contrast, our case involved the mitral valve and presented with an embolic stroke complicated by splenic infarction. The treatment for embolic stroke and splenic infarction included IV linezolid, eravacycline, and ampicillin. Due to the challenges of closely monitoring the patient at a skilled nursing facility while receiving linezolid, which necessitates frequent laboratory follow-ups, eravacycline was introduced as an additional medication. This decision was influenced by positive efficacy data demonstrating its effectiveness against VRE [8].

It was determined that our patient was not a surgical candidate based on clinical evaluations by cardiothoracic surgery. In addition, the patient’s family did not desire for her to undergo any form of surgery. In general, surgical indications for endocarditis are related to heart failure or shock, evidence or risk of persistent infection, and embolic risk reduction. The European Society of Cardiology recommends urgent surgery for aortic or mitral valve vegetation >10 mm with severe stenosis or regurgitation and low operative risk [9]. Furthermore, age and hemodynamic instability are two of the strongest predictors of operative risk. The American College of Cardiology recommends that when a hemorrhagic stroke or extensive neurologic damage is present, surgery should be delayed by more than four weeks [9].

Other cases of IE with multiple embolic locations not in the setting of VRE have been reported. Atilla et al. presented a case of a 47-year-old man on hemodialysis who developed IE on the mitral valve complicated by splenic and cerebral infarction and was treated with intravenous teicoplanin, gentamicin, subcutaneous low-molecular-weight heparin, and oral acetylsalicylic acid. When the patient improved clinically, mitral valve replacement was performed [1]. 

Treatment guidelines involving IE with multiple embolic locations are on a case-to-case basis and require a multidisciplinary, multimodal therapeutic approach. Broad-spectrum antibiotic therapy is the most important initial step in the management of IE. Once the presence of an organism is confirmed, antibiotics should be optimized according to sensitivity [3]. Specifically, for VRE, linezolid has been shown to be an effective medication [10]. However, its prolonged use has been associated with thrombocytopenia, anemia, and peripheral neuropathy [11]. Another medication commonly prescribed for VRE is tigecycline, which has good coverage against gram-positives, gram-negatives, and anaerobes [4]. Resistance to tigecycline in VRE has not been reported yet [4]. Currently, no data exist regarding the use of tigecycline in the treatment of IE due to VRE. Further treatment may be guided by the type and location of emboli. Vascular or endovascular interventions for arterial aneurysms, percutaneous drainage of abscesses, or organ resection such as splenectomy for infarctions may be considered as well.

## Conclusions

Given the high mortality rates associated with VRE endocarditis and the increasing prevalence of antibiotic-resistant infections, there is a need for continued research to better understand risk factors and optimize management strategies for this condition. This case report serves as a reminder to clinicians to maintain a high level of suspicion for VRE in patients with IE.
